# Enhancing Nutrition Communication in Early Childhood Education Settings: Overcoming Challenges and Promoting Collaboration with Caregivers

**DOI:** 10.3390/ijerph22050677

**Published:** 2025-04-25

**Authors:** Elder G. Varela, Ciana Bonfiglio, Jamie Zeldman, Alexandra Chavez, Amy R. Mobley

**Affiliations:** 1 Department of Health Education and Behavior, University of Florida, Yon Hall North, Room 033, Gainesville, FL 32611, USA; egvarela@umich.edu (E.G.V.); cianajbonfiglio@gmail.com (C.B.); jzeldman@ufl.edu (J.Z.); alexandrachavez@usf.edu (A.C.); 2 Department of Nutritional Sciences, School of Public Health, University of Michigan, 1415 Washington Heights, Ann Arbor, MI 48109, USA; 3 Wellness Dietitian, Recreation and Wellness, University of North Florida, Jacksonville, FL 32224, USA; 4 Morsani College of Medicine, University of South Florida, Tampa, FL 33602, USA

**Keywords:** child day care centers, early childhood providers, nutrition strategies, pediatric obesity

## Abstract

Early childhood plays a critical role in shaping food preferences and eating habits, emphasizing the importance of effective communication between families and Early Childhood Education (ECE) settings to promote healthy eating behaviors. This qualitative study examines ECE providers’ perspectives on barriers and facilitators to nutrition communication with caregivers, comparing Early Head Start/Head Start (EHS/HS) and non-EHS/HS centers. Semi-structured interviews were conducted via Zoom with ECE providers (*n* = 20) serving children aged 0–3 in Florida. Using inductive thematic analysis, two researchers independently coded the data, identified themes, and compared similarities and differences between EHS/HS and non-EHS/HS providers through an iterative review process. Participants were predominantly white (74%) females (95%) with an average of 7.6 years of work experience. Both EHS and non-EHS providers reported common barriers, including limited time, caregiver resistance to change, and language challenges. However, EHS providers identified additional issues like economic constraints, limited nutrition knowledge among both caregivers and providers, and restricted access to technology. In contrast, non-EHS providers emphasized trust issues and caregiver non-compliance with center policies. To improve communication, EHS providers suggested trust-building and documenting dietary intake for personalized interactions, while non-EHS providers recommended regular meetings and mobile messaging. Addressing these barriers is crucial for fostering collaboration between providers and caregivers and promoting healthy food habits in young children.

## 1. Introduction

Childhood obesity in the United States has reached critical levels, with nearly 12.7% of children aged 2 to 5 classified as obese as of 2023 [1]. This period, when food preferences and eating behaviors are typically formed, is heavily influenced by primary caregivers and early childhood educators [2,3,4]. With more than two-thirds of American children having parents who are employed full-time, childcare plays a fundamental role not only in early learning and development but also in enabling parents to pursue work, education, or job training [5]. In 2019, children spent an average of over 183 h per month in childcare settings, where they receive multiple meals and snacks daily [6].

During the critical developmental stage of ages 2–5, children undergo rapid physical growth, cognitive development, and refinement of motor skills [7]. Proper nutrition is essential to support brain development, immune function, and overall growth [7]. Additionally, this stage marks the beginning of forming lifelong eating habits, and a positive eating environment can significantly influence future health outcomes [8]. Parenting functions, such as modeling healthy eating behaviors, offering appropriate portion sizes, and fostering self-regulation skills, are pivotal in shaping children’s nutrition [9,10,11]. Outside the home, caregiving environments like early childhood settings (i.e., childcare centers and preschools) also play a critical role in influencing children’s food choices, mealtime routines, and exposure to various foods, specifically healthy, nutrient-rich options, which can reinforce or challenge the nutrition habits established at home [12,13,14].

Given the significant time children spend in these environments, collaboration between caregivers/parents and Early Childhood Education (ECE) providers is essential for promoting healthy growth and preventing obesity [15,16]. Effective communication—through daily updates, workshops, newsletters, and digital platforms—helps ensure consistent nutrition messaging across settings [17]. Childcare providers play a key role in educating parents on child nutrition through regular information sharing. While time constraints and cultural differences can pose challenges, mobile apps have been found to facilitate effective communication, ultimately enhancing parental engagement and supporting children’s dietary outcomes [18].

The Social Cognitive Theory (SCT) provides a valuable framework for examining the factors influencing ECE providers’ nutritional communication practices with caregivers of infants and toddlers [19]. The SCT posits that human behavior is shaped by the dynamic interplay of personal, behavioral, and environmental factors, emphasizing reciprocal determinism—the idea that behavior is influenced by the simultaneous interaction of individual characteristics, actions, and surroundings [19]. In this context, the individual characteristics of both ECE providers and primary caregivers (e.g., parents and legal guardians) significantly influence their perspectives on nutrition and food choices. For ECE providers, factors such as education level, nutrition training, personal beliefs about healthy eating, and cultural backgrounds shape their communication of nutritional practices, while similar factors affect caregivers’ attitudes and support for healthy eating at home.

Effective communication between ECE providers and caregivers/parents is, therefore, critical, encompassing discussions on meal planning and sharing resources like recipes and educational materials. Both groups promote positive mealtime practices, with ECE providers encouraging healthy behaviors in the classroom and parents reinforcing them at home. Additionally, the surroundings of ECE settings, including the availability of healthy food options and a supportive atmosphere for discussing nutrition, further impact these interactions. Caregivers can also influence this environment by providing nutritious food and fostering family mealtime routines at home. Understanding these interconnected factors can enhance communication strategies between ECE providers and parents, ultimately improving children’s nutritional outcomes.

Despite the presence of specific nutrition-related policies and regulations for private and public childcare settings, there needs to be a noticeable gap in promoting adequate nutrition communication between ECE providers and caregivers [15,20,21]. This study aims to explore ECE providers’ perceptions of the barriers and opportunities that contribute to effective nutrition communication. Furthermore, this study aims to compare the perceptions of nutrition communication practices between providers in Early Head Start (EHS) programs and those in non-Early Head Start (non-EHS) programs. EHS programs primarily serve low-income families and follow more structured, federally mandated nutrition and family engagement guidelines [22], while non-EHS programs may not have the same level of federal oversight or specific focus on low-income populations, offering more flexibility in their approach to nutrition and family engagement. Comparing EHS and non-EHS programs is crucial, as the federally mandated guidelines in EHS settings likely shape different approaches to communication between ECE providers and caregivers. By examining these differences, this study provides insight into the unique barriers and opportunities in each setting, supporting tailored recommendations to improve nutrition practices and strengthen caregiver-provider communication across diverse ECE contexts.

## 2. Materials and Methods

### 2.1. Study Design

This qualitative study aims to explore the challenges and supportive factors that influence Early Childhood Education (ECE) providers’ communication about nutrition with caregivers. Semi-structured interviews were chosen to allow ECE providers to share personal insights in a comfortable and confidential setting, avoiding the potential influence of group dynamics. Interviews were conducted with ECE providers from both Early Head Start (EHS) and non-EHS centers, offering a comparison of program-specific barriers and facilitators. The study design focused on obtaining in-depth, individualized perspectives on nutrition-related communication in Early Childhood Education settings.

### 2.2. Informant Recruitment

A convenience sample of ECE providers, defined as teachers or staff members responsible for the care and education of children aged 0–3, was recruited from both EHS and non-EHS centers. To be eligible, participants needed to have a minimum of three years of experience working with young children and regular interactions with caregivers, such as parents or legal guardians. Recruitment strategies included distributing flyers at ECE centers, sending email announcements to potential participants, and following up with phone calls. Up to two ECE providers per center were permitted to participate to ensure a balanced representation across multiple centers without concentrating responses from a single site. Each participant received a USD 20 electronic gift card as an incentive for their time and participation. The University of Florida’s Institutional Review Board for Human Subjects reviewed and approved all study procedures.

### 2.3. Data Collection

Data collection consisted of semi-structured interviews conducted via Zoom, allowing flexibility and reducing logistical challenges for participants. Interviews lasted approximately 30 to 45 min, providing sufficient time to explore the topics in depth while respecting participants’ time constraints. Before each interview, participants were informed about the study’s purpose, and written informed consent was obtained. All interviews were audio-recorded with participants’ explicit permission, and recordings were transcribed verbatim. Following the interviews, participants completed a brief demographic questionnaire via Qualtrics, a secure and widely used online survey platform, capturing information on their age, gender, professional background, and years of experience. The recorded interviews were securely stored; audio files were encrypted and stored in a password-protected folder with restricted access to ensure data protection and confidentiality in accordance with General Data Protection Regulation (GDPR) guidelines.

### 2.4. Interview Guideline

An interview guide was developed based on SCT constructs, specifically focusing on perceived opportunities and barriers, behavioral capability, and self-efficacy [23]. Questions aimed to identify individual and systemic factors that affect ECE providers’ ability to communicate effectively about nutrition. For example, one question asked: “What are some of the challenges or barriers that may interfere with your ability to communicate with caregivers about child nutrition?”. Additionally, the guide assessed providers’ confidence and skills in discussing nutrition topics and their views on the role and responsibility of ECE providers in supporting healthy eating practices in ECE settings. For instance, one question asked: “On a scale of 1 to 5, how confident do you feel about communicating with caregivers about child nutrition information? (1 = not at all confident, 5 = very confident) Why?”. This guide was developed by the authors [EGV, CB, and ARM], who have extensive experience in qualitative research, early childhood nutrition, and health behavior. Before implementation, the guide was tested by a childcare professional to ensure the clarity and relevance of all seven questions. This testing allowed for refinement based on feedback received during the preliminary interviews, ensuring the guide was well-suited to the study’s objectives.

### 2.5. Data Analysis

Quantitative data from the demographic questionnaire were analyzed using descriptive statistics in IBM SPSS Statistics 29 to summarize participant characteristics [24]. Content analysis was conducted on the qualitative data using an inductive coding approach [19], allowing themes to emerge directly from the data without predetermined hypotheses. This method ensured that the analysis accurately reflected participants’ experiences and perspectives [25]. The coding process followed established qualitative research procedures, beginning with an open coding phase where two trained research assistants [EGV and CB] independently reviewed and coded the transcripts. The research team systematically organized codes into broader categories through an iterative process of discussion and refinement. To enhance reliability, coders met regularly to compare codes, resolve discrepancies, and achieve consensus on final themes. Data from EHS and non-EHS providers were analyzed separately to identify program-specific differences. A cross-tabulated matrix was then developed to systematically compare emergent themes across program types, facilitating an in-depth examination of variations in communication barriers and facilitators. Data collection continued until thematic saturation was reached, ensuring that no new themes emerged from additional interviews.

## 3. Results

The demographic characteristics of the participating ECE providers (*n* = 20) are reported in Table 1. Overall, participants were primarily white (74%) females (95%) with an average of 7.6 years of experience working in their current ECE setting. Participants reported that they had an average of 53 children enrolled at their centers.

The following section outlines the themes that emerged from ECE providers’ perspectives on communicating about nutrition with caregivers. Table 2 provides a summary of these themes, detailing both barriers and strategies along with illustrative quotes.

### 3.1. Barriers to Effective Provider-Caregiver Nutrition Communication Practices

Similar themes emerged across both EHS and non-EHS providers, underscoring shared challenges in achieving effective nutrition communication with caregivers. A primary barrier perceived by ECE providers was the limited time available for meaningful discussions with caregivers. Participants noted that the brief interactions at drop-off and pick-up often left little opportunity to engage deeply in feeding behaviors and healthy eating practices. Additionally, participants noted a reluctance among some caregivers to alter established nutrition routines. ECE providers perceived that many caregivers, feeling their parenting choices were under scrutiny, could become defensive, further restricting open dialogue regarding children’s dietary habits.

ECE providers also identified language differences as a key barrier, especially with non-English-speaking caregivers. Participants reported that these language obstacles often hindered effective comprehension and engagement, as translation tools sometimes inaccurately conveyed information, leading to misunderstandings. Additionally, providers observed that economic challenges faced by caregivers potentially restricted their access to nutritious foods, influencing both their openness to nutritional guidance and their perceived capacity to act on it.

Distinct differences emerged between the groups based on the type of center. Providers in EHS centers emphasized the lack of nutrition training as a significant barrier to delivering effective guidance, expressing concerns that both they and the caregivers had not received adequate education on nutritional best practices. This perceived gap in knowledge left providers feeling ill-equipped to address nutrition-related questions and concerns. In contrast, non-EHS providers highlighted trust as a fundamental issue impacting their communication with caregivers. They noted that the absence of strong, trusting relationships made it challenging to initiate sensitive conversations about nutrition; without established rapport, caregivers were less likely to engage openly or consider recommendations from providers.

### 3.2. Strategies for Improving Nutrition Communication Practices

In response to these barriers, ECE providers working at both EHS and non-EHS centers shared strategies they believed could improve nutrition communication with caregivers. A common theme among ECE providers was the need for enhanced training to equip them with the knowledge necessary for effective communication about nutrition. ECE providers suggested training or workshops focused on nutrition education, covering topics such as strategies for addressing picky eating, managing food allergies, and understanding dietary guidelines. Lastly, ECE providers from both EHS and non-EHS centers advocated for stronger, evidence-based nutrition policies to create a supportive dietary environment for children. Examples such as standardized menus prioritizing whole grains, fruits, and vegetables, nutrition education resources for caregivers, guidelines for portion sizes and healthy snacks, and promoting family-style meals to encourage food exploration were among their recommendations.

Different recommendations also emerged between ECE provided in EHS and non-EHS settings utilizing documentation, such as weekly reports, to share children’s dietary intake with caregivers, viewing this as a practical way to personalize communication. They believed that maintaining detailed records facilitated more targeted discussions about children’s eating behaviors, enabling providers to offer tailored guidance and support. Conversely, participants in non-EHS settings emphasized the importance of structured, consistent meetings with caregivers as essential for establishing regular touchpoints to address nutrition-related concerns. They felt these meetings could provide opportunities for ongoing dialogue and relationship building. Specifically, ECE providers at EHS highlighted the importance of building trustful relationships with caregivers as essential for creating an open and supportive environment for nutrition discussions. However, they did not provide specific strategies for achieving this goal.

Table 2 summarizes these themes, displaying barriers and strategies alongside quotes that reflect the perspectives shared by providers.

## 4. Discussion

This study highlights the essential role that ECE providers play in promoting healthy eating behaviors among young children and fostering effective nutrition communication with caregivers. ECE providers not only influence children’s early eating habits but also serve as important sources of nutrition guidance for caregivers [15,16]. Nevertheless, this study underscores that despite the recognition of this vital role, significant barriers prevent ECE providers from fully engaging in meaningful, impactful nutrition discussions with caregivers. These barriers include a lack of nutrition knowledge, limited time for discussions, and, notably, a lack of trust and open communication between childcare providers and caregivers, which impede the development of productive, collaborative relationships.

Consistent with previous research, ECE providers encounter multiple challenges in nutrition communication, such as time constraints that limit opportunities for in-depth discussions on children’s nutrition, as well as caregivers’ reluctance to alter their established feeding practices [15,16,26]. These issues are compounded by a general lack of training in child nutrition among ECE providers, which often leaves them feeling ill-equipped to initiate or lead nutrition-focused conversations with caregivers [15,16,26]. This dynamic is further complicated by caregivers’ tendency to view ECE centers primarily as care providers rather than collaborative partners in children’s overall well-being [14]. As such, many caregivers do not see value in engaging in nutrition-related discussions with ECE providers or may not fully appreciate the role these discussions could play in shaping their child’s health.

Moreover, caregivers often resist conversations about their children’s eating habits, particularly when these discussions challenge their established practices. This resistance can stem from feelings of defensiveness, as caregivers may perceive suggestions regarding their children’s diets as criticism or judgment. These perceptions can create tension and hinder open, constructive dialogues about nutrition, further complicating efforts to collaborate on improving children’s eating habits. This aligns with previous studies that have highlighted the difficulty of initiating nutrition-related conversations when caregivers view these conversations as unnecessary or feel that their parenting choices are being questioned or criticized [26].

Despite these challenges, ECE providers identified several potential strategies to enhance communication with caregivers. Notably, these strategies include provider training in nutrition, utilizing electronic resources to bridge the communication gap, and fostering collaborative partnerships with caregivers. However, this study also emphasizes that such strategies need to be tailored to address the unique challenges faced by different types of ECE providers, particularly those in Early Head Start centers. EHS providers may face different structural and resource-related constraints compared to non-EHS providers, which could influence their ability to engage in effective nutrition communication.

A key strength of this study lies in its unique contribution to understanding the different experiences of EHS and non-EHS providers in nutrition communication. HS providers identified barriers such as a lack of nutrition education and training for caregivers and limited financial resources, which hinder their ability to support healthy eating practices. In contrast, non-EHS providers highlighted issues related to lack of trust and open relationships with caregivers, as well as caregivers’ provision of unhealthy foods. These findings underscore the need for targeted, context-specific interventions that address the unique barriers faced by each group, enhancing the effectiveness of nutrition communication and improving children’s nutritional outcomes. These findings extend previous research that underscores the critical role of ECE providers as nutrition gatekeepers, highlighting the need for interventions that address both the practical and relational challenges that providers encounter in their work [15,16].

Despite its strengths, this study has limitations. The sample may not fully represent the experiences of all ECE providers, and self-reported data may introduce bias, particularly in terms of caregivers’ willingness to openly discuss barriers or concerns. Additionally, the Coronavirus disease (COVID-19) pandemic may have influenced participants’ perspectives on communication barriers and practices, as many ECE providers had to adjust to new, virtual methods of engaging with caregivers. These factors should be considered when interpreting the study’s findings, and they point to the need for future research that explores how these challenges have evolved over time, particularly in the context of a rapidly changing social and health landscape.

Future research should continue to explore strategies for mitigating the barriers identified in this study and enhancing nutrition communication in ECE settings. Longitudinal studies examine the impact of interventions—such as specialized provider training or the introduction of digital tools for communication—that could offer valuable insights into the long-term effectiveness of these strategies. Additionally, research that investigates the role of how contextual factors, such as program type (EHS vs. non-EHS), organizational support, and community resources, would provide a deeper understanding of the factors that influence the success of nutrition communication efforts. Expanding the sample to include a more diverse range of ECE providers, as well as incorporating observational data, could provide a more comprehensive understanding of the challenges and opportunities in promoting healthy eating behaviors among young children. Furthermore, incorporating the perspectives of caregivers themselves would help bridge the gap between provider recommendations and caregiver perceptions, ultimately leading to more effective and collaborative nutrition education strategies.

## 5. Conclusions

This study offers insights into the barriers and opportunities for improving nutrition communication between ECE providers and caregivers. Findings from this study underscore the importance of addressing the multifaceted barriers to nutrition communication in ECE settings. By focusing on the distinct needs of EHS and non-EHS providers, developing targeted training programs, and utilizing digital tools to support communication, it is possible to enhance the partnership between providers and caregivers. This collaboration can help overcome existing challenges and ensure that young children receive the nutrition education and support they need to develop healthy eating habits that will benefit them throughout their lives. Future research should also focus on understanding caregivers’ perspectives and developing targeted educational materials and training programs.

## Figures and Tables

**Table 1:** Demographic characteristics of Early Childhood Education providers (*n* = 20).

Demographic Characteristics	Overall (*n* = 20)	EHS (*n* = 10)	Non-EHS (*n* = 10)
Age (y), mean (SD)	44.2 (15.1)	48.3 (15.4)	38.4 (13.8)
Race/Ethnicity, *n* (%)			
White	14 (74)	9 (90)	5 (56)
Black or African American	1 (5)	1 (10)	-
Am Indian/Alaskan Native	1 (5)	-	1 (11)
Hispanic	3 (16)	-	3 (33)
Sex, *n* (%)			
Female	19 (95)	10 (100)	9 (90)
Male	1(5)	-	1 (10)
Highest level of education, *n* (%)			
High school graduate	1(5)	1 (10)	-
Some college	2 (10)	1 (10)	1 (10)
Associate’s degree/technical school	6 (30)	4 (40)	2 (20)
Baccalaureate degree	6 (30)	2 (20)	4 (40)
Advanced college degree	5 (25)	2 (20)	3 (30)
Center Characteristics			
Number of children enrolled, mean (SD)	53.3 (30.0)	49.6 (22.6)	56.7 (36.4)
Length of time position, y, mean (SD)	7.6 (6.4)	6.7 (7.2)	8.4 (5.8)
Receive Childcare Food Program, *n* (%)	11 (55)	7 (70)	4 (40)

Note: Values are expressed as mean (SD) or median (25th and 75th percentiles) for continuous variables and as absolute numbers (*n*) and frequencies (%) for categorical variables.

**Table 2:** Perceived Barriers and Strategies for Effective Nutrition Communication Between ECE Providers Working at EHS and Non-EHS Settings.

Theme	Sub-Themes	ECE Providers at EHS	ECE Providers at Non-EHS
Barriers to Effective Nutrition Communication Practices	Lack of Time (i.e., limited availability or competing responsibilities that prevent engagement between caregivers and providers)	“There’s just no time to talk to parents during the day.” (P1-EHS)	“Drop-off interactions are very quick, sometimes just a few seconds.” (P1-N-EHS)
	Lack of Caregiver Receptiveness (i.e., defensive reactions, feeling attacked, or perceiving feedback as criticism rather than constructive guidance)	“Parents get defensive or hurt, thinking we’re criticizing their parenting.” (P2-EHS)	“Parents are our customers, and we can’t tell them how to parent.” (P2-N-EHS)
	Language Barriers (i.e., difficulties in communication due to differences in language proficiency)	“We use Google Translate for Spanish-speaking parents, but it’s not perfect.” (P3-EHS)	“Even with a translator, important information can be missed.” (P3-N-EHS)
	Caregivers’ Resistance to Change (i.e., reluctance or resistance to adopting recommended nutrition practices or incorporating feedback about children’s dietary habits)	“There’s cultural differences in food preferences, and parents are often set in their ways.” (P4-EHS)	“Drop-off interactions are very quick, sometimes just a few seconds.” (P1-N-EHS)
	Lack of Nutrition Education and Training (i.e., insufficient knowledge or skills among caregivers to effectively support children’s healthy eating habits and nutrition-related practices)	“Parents often find it easier to give unhealthy foods because they lack the knowledge.” (P5-EHS)	Not mentioned
	Caregivers’ Lack of Financial Means (i.e., limited financial resources that restrict access to healthy food options)	“Some parents may not afford nutritional foods, making it hard to follow recommendations.” (P6-EHS)	Not mentioned
	Lack of Trust and Open Relationship with Caregivers (i.e., insufficient communication or rapport between caregivers and providers, leading to skepticism and reluctance to engage)	Not mentioned	“Having a good relationship with parents is crucial for honest conversations about nutrition.” (P5-N-EHS)
	Caregivers’ Provision of Unhealthy Foods (i.e., offering energy-dense, nutrient-poor foods, such as sugary snacks and processed foods)	Not mentioned	“Parents sometimes provide unhealthy foods like candy for breakfast.” (P6-N-EHS)
Strategies for Improving Nutrition Communication Practices	Establishing Regular Meetings with Caregivers (i.e., setting up consistent communication through scheduled meetings)	“We try to make it a habit to reach out to families regularly.” (P7-EHS)	“We should standardize phone calls or parent meetings to discuss nutrition.” (P7-N-EHS)
	Sending Targeted Messages Electronically (i.e., using digital platforms, such as emails, texts, or apps, to deliver personalized information)	“We send messages on Learning Genie to keep parents informed.” (P8-EHS)	“Using apps or social media can help catch parents’ attention about nutrition topics.” (P8-N-EHS)
	Providing Nutrition Education and Training Materials (i.e., offering resources such as brochures, online courses, workshops, or guides to enhance caregivers’ knowledge of healthy eating practices)	“We need more updated literature and resources to give to parents.” (P9-EHS)	“We try to communicate healthy recipes or activities that parents can do at home.” (P9-N-EHS)
	Building Open, Positive, and Trusting Relationships with Caregivers (i.e., fostering transparent, empathetic communication, offering support without judgment, and creating a safe space for caregivers)	“Good relationships with parents encourage open communication about their child’s nutrition.” (P10-EHS)	Not mentioned
	Documenting Children’s Dietary Intake (i.e., tracking and recording children’s food consumption through observational logs to monitor dietary patterns)	“We keep a log of what children eat and inform parents regularly.” (P11-EHS)	Not mentioned
	Adopting and Implementing Policies Related to Evidence-Based Dietary Recommendations (i.e., integrating established nutrition guidelines and strategies into organizational practices and policies)	Not mentioned	“We enforce policies to reduce sugar intake in the foods provided.” (P10-N-EHS)

## Data Availability

The raw data supporting the conclusions of this article will be made available by the authors on request.

## Abbreviations

The following abbreviations are used in this manuscript:

SCT	Social Cognitive Theory
ECE	Early Childhood Education
EHS	Early Head Start
Non-EHS	Non-Early Head Start
COVID-19	Coronavirus disease

## References

[1] Centers for Disease Control and Prevention (2024). Childhood Obesity Facts. https://www.cdc.gov/obesity/childhood-obesity-facts/childhood-obesity-facts.html.

[2] Lindsay A.C., Sussner K.M., Kim J., Gortmaker S.L. (2006). The Role of Parents in Preventing Childhood Obesity. Future Child..

[3] Natale R., Scott S.H., E Messiah S., Schrack M.M., Uhlhorn S.B., Delamater A. (2013). Design and methods for evaluating an early childhood obesity prevention program in the childcare center setting. BMC Public Health.

[4] Natale R., Uhlhorn S.B., Lopez-Mitnik G., Camejo S., Englebert N., Delamater A.M., Messiah S.E. (2016). Caregiver’s Country of Birth Is a Significant Determinant of Accurate Perception of Preschool-Age Children’s Weight. Health Educ. Behav..

[5] Schneider A., Gibbs H. (2023). Data Dashboard: An Overview of Child Care and Early Learning in the United States.

[6] National Center for Education Statistics (2021). Fast Facts: Child Care.

[7] Black M.M. (2018). Impact of Nutrition on Growth, Brain, and Cognition. Nestlé Nutr. Inst. Workshop Ser..

[8] Martín-Rodríguez A., Bustamante-Sánchez Á., Martínez-Guardado I., Navarro-Jiménez E., Plata-SanJuan E., Tornero-Aguilera J.F., Clemente-Suárez V.J. (2022). Infancy Dietary Patterns, Development, and Health: An Extensive Narrative Review. Children.

[9] Schratz L.M., Larkin O., Dos Santos N., Martin C. (2023). Caregiver Influences on Eating Behaviors in Children: An Opportunity for Preventing Obesity. Curr. Atheroscler. Rep..

[10] Mahmood L., Flores-Barrantes P., Moreno L.A., Manios Y., Gonzalez-Gil E.M. (2021). The Influence of Parental Dietary Behaviors and Practices on Children’s Eating Habits. Nutrients.

[11] Chen B., Kattelmann K., Comstock C., McCormack L., Wey H., Meendering J. (2021). Parenting Styles, Food Parenting Practices and Dietary Intakes of Preschoolers. Nutrients.

[12] Ward D.S., Welker E., Choate A., Henderson K.E., Lott M., Tovar A., Wilson A., Sallis J.F. (2017). Strength of obesity prevention interventions in early care and education settings: A systematic review. Prev. Med..

[13] Arlinghaus K.R., Schroeder M., Gold A., Goodman L.P., Wagner G., Pass M., Hunt S., Stang J. (2023). Outcomes From Healthy Eating and Physical Activity Recognition Programs in Early Child Care and Education: A Scoping Review. Am. J. Health Promot..

[14] Lanigan J.D. (2012). The relationship between practices and child care providers’ beliefs related to child feeding and obesity prevention. J. Nutr. Educ. Behav..

[15] Dev D.A., Byrd-Williams C., Ramsay S., McBride B., Srivastava D., Murriel A., Arcan C., Adachi-Mejia A.M. (2017). Engaging Parents to Promote Children’s Nutrition and Health. Am. J. Heal. Promot..

[16] Garcia A.S., Dev D.A., Stage V.C. (2018). Predictors of Parent Engagement Based on Child Care Providers’ Perspectives. J. Nutr. Educ. Behav..

[17] Xu T., Akerson A., Fox B. (2021). Incorporating technology to foster effective communication and documentation in child care programs. Educ. Inf. Technol..

[18] Dev D.A., Garcia A.S., Dzewaltowski D.A., Sisson S., Franzen-Castle L., Rida Z., Williams N.A., Hillburn C., Dinkel D., Srivastava D. (2020). Provider reported implementation of nutrition-related practices in childcare centers and family childcare homes in rural and urban Nebraska. Prev. Med. Rep..

[19] Glanz K., Rimer B.K., Viswanath K. (2015). Health Behavior: Theory, Research, and Practice.

[20] Lyn R., Evers S., Davis J., Maalouf J., Griffin M. (2014). Barriers and Supports to Implementing a Nutrition and Physical Activity Intervention in Child Care: Directors’ Perspectives. J. Nutr. Educ. Behav..

[21] Mena N.Z., Risica P.M., Gans K.M., Lofgren I.E., Gorman K., Tobar F.K., Tovar A. (2020). Communication With Family Child Care Providers and Feeding Preschool-Aged Children: Parental Perspectives. J. Nutr. Educ. Behav..

[22] Administration for Children & Families: Office of Head Start (2021). Head Start History. https://www.acf.hhs.gov/ohs/about/history-head-start.

[23] Braun V., Clarke V. (2006). Using thematic analysis in psychology. Qual. Res. Psychol..

[24] IBM Corp (2020). IBM SPSS Statistics for Windows, version 27.0.

[25] Azungah T. (2018). Qualitative research: Deductive and inductive approaches to data analysis. Qual. Res. J..

[26] Johnson S.L., Ramsay S., Shultz J.A., Branen L.J., Fletcher J.W. (2013). Creating Potential for Common Ground and Communication Between Early Childhood Program Staff and Parents About Young Children’s Eating. J. Nutr. Educ. Behav..

